# A twig-like insect stuck in the Permian mud indicates early origin of an ecological strategy in Hexapoda evolution

**DOI:** 10.1038/s41598-021-00110-2

**Published:** 2021-10-21

**Authors:** Antoine Logghe, André Nel, Jean-Sébastien Steyer, Valérie Ngô-Muller, Jean-Marc Pouillon, Romain Garrouste

**Affiliations:** 1 Centre de Recherches en Paléontologie – Paris, UMR 7207 – CNRS, MNHN, SU, Muséum National d’Histoire Naturelle, 8 rue Buffon, CP38, 75005 Paris, France; 2 Institut de Systématique, Évolution, Biodiversité, ISYEB-UMR 7205-CNRS, MNHN, UPMC, EPHE, Muséum National d’Histoire Naturelle, Sorbonne Universités, Université des Antilles, 57 rue Cuvier, CP 50, Entomologie, 75005 Paris, France; 3 Faculté des Sciences, UFR Sciences du Vivant, Université de Paris, 5, rue Thomas Mann, 75013 Paris, France; 4 Rhinopolis, 179 rue des Plattières, 38300 Nivolas Vermelle, France

**Keywords:** Evolution, Palaeontology

## Abstract

Full body impressions and resting traces of Hexapoda can be of extreme importance because they bring crucial information on behavior and locomotion of the trace makers, and help to better define trophic relationships with other organisms (predators or preys). However, these ichnofossils are much rarer than trackways, especially for winged insects. Here we describe a new full-body impression of a winged insect from the Middle Permian of Gonfaron (Var, France) whose preservation is exceptional. The elongate body with short prothorax and legs and long wings overlapping the body might suggests a plant mimicry as for some extant stick insects. These innovations are probably in relation with an increasing predation pressure by terrestrial vertebrates, whose trackways are abundant in the same layers. This discovery would possibly support the recent age estimates for the appearance of phasmatodean-like stick insects, nearly 30 million years older than the previous putative records. The new exquisite specimen is fossilized on a slab with weak ripple-marks, suggesting the action of microbial mats favoring the preservation of its delicate structures. Further prospections in sites with this type of preservation could enrich our understanding of early evolutionary history of insects.

## Introduction

Some outcrops are clearly more favourable for the preservation of trackways, resting traces and full body impressions than for the organisms themselves. The trackways of arthropods are rather frequent in the fossil record since the Devonian. But they are especially frequent in the continental outcrops of the ‘red’ Permian in Europe and North America^1–3^. Nevertheless, the trackmakers are generally difficult to determine, especially for those attributed to arthropods^4^. The resting traces and the full-body impressions (FBIs) are more complex than the trackways and give more information on the external morphology of the trackmakers. FBIs can be nearly as well-preserved as a body fossils^5^. But these are much rarer than the trackways, and among them, those attributed to the Hexapoda are even the rarest, mainly attributed to apterous Archaeognatha (Supplementary Information)^5–11^. FBIs of winged insects are extremely rare: only 10 specimens are recorded in the world, from the Carboniferous and Permian (Table 1 in Supplementary Information) and they mostly preserve the ventral side of the trackmaker only.

Here we describe an exquisite full-body impression of an elongate winged insect strongly resembling a Phasmatodea (stick insect), from the Middle Permian of the Luc Basin (Var, Provence, France). It consists in a delicate lateral impression of the entire insect. With only few occurrences from the Middle Triassic to the Cenozoic, the fossil record of the Phasmatodea is very poor compared to that of the other Polyneoptera such as the Orthoptera^12–18^. The earliest occurrences of stick insects are scarce and based on incomplete bodies or wing fossils^14,17–20^.

Stick insects are one of the most specialised insect orders in term of plant mimicry. New discoveries about their early origins give clue to the development of such a defence strategy, currently known from only a few fossil taxa^16,21–24^. Body fossils of Phasmatodea remain unknown from the Palaeozoic. The present discovery would be the oldest evidence of a potential phasmatodean-like insect, suggesting the stick insects were already present more than 30 million years than though through other records, during the Mesozoic^14,17^. Independently to the taxonomic attribution of this FBI that can be only tentative by nature, the general shape of its narrow and elongate body strongly resembles that of a plant twig. Thus, it would also possibly constitutes the second oldest case of plant mimicry by insects, the first one being based on a leaf mimicry by an Orthoptera Tettigonioidea from the Roadian (Middle Permian) of Dôme du Barrot (France)^24^.

urn:lsid:zoobank.org:pub:985D009E-7D62-4BA4-8245-55E894563F64.

## Results

### Systematic paleoichnology

Ichnogenus Phasmichnus gen. nov.

- urn:lsid:zoobank.org:act:0D2B8221-402F-4C7D-A8EF-D28BA690C857
- *Type species. Phasmichnus radagasti* sp. nov.
- *Diagnosis.* Lateral impression composed of three tagmata, the median bearing three legs. Very elongated and narrow median and posterior tagmata. Presence of long traces of putative wings whose base is behind hind legs. Abdomen with traces of terminal appendages (cerci and/or ovipositor).
- *Etymology.* After Phasmatidae Gray, 1835 and ichnos (Greek for trace).
- *Composition and distribution*. Only *Phasmichnus radagasti* sp. nov. (Middle Permian, France) (see Supplementary Fig. 1 and Supplementary Information).

### *Phasmichnus radagasti* sp. nov. (Figs. 1, 2)

**Figure 1:**
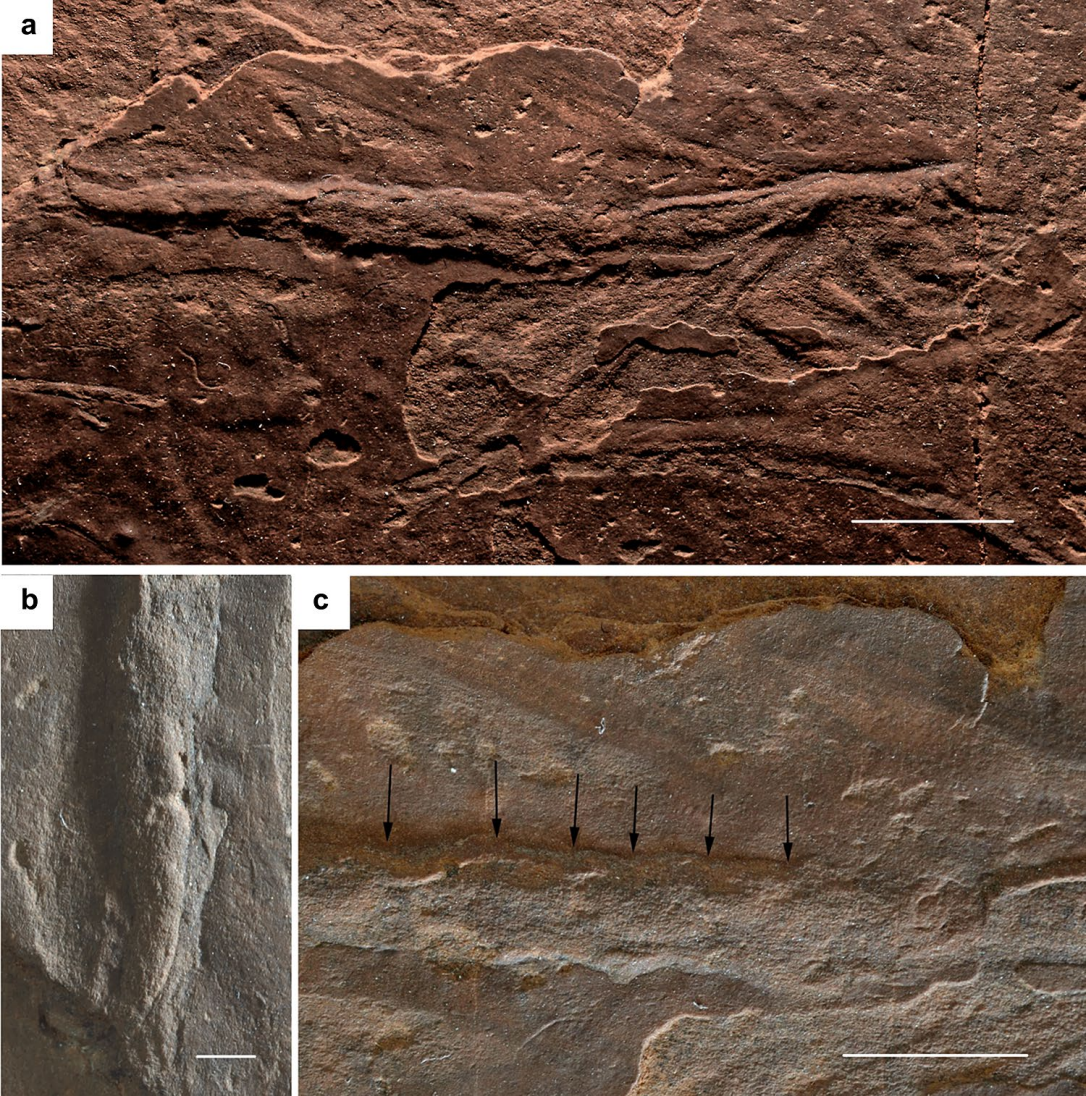
*Phasmichnus radagasti* nov. gen. et nov. sp., holotype MNHN.F.A71341. (**a**) Photograph of general habitus. (**b**) Close-up on apex of abdomen. (**c**) Close-up on abdomen base (arrows: putative lateral limits of segments). Photographs under natural light by V.N.-M. Scale bars, 10 mm (**a**), 5 mm (**b**), 1 mm (**c**).

**Figure 2:**
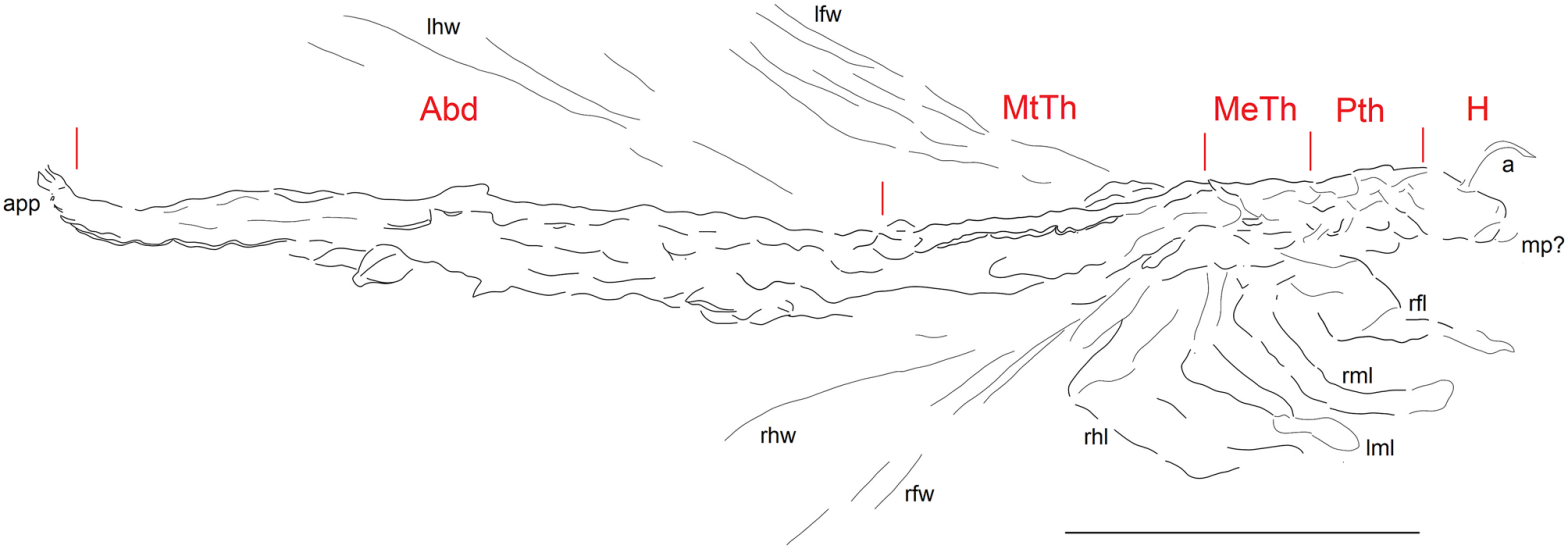
*Phasmichnus radagasti* gen. et nov. sp. Interpretative drawing. Abd abdomen; a antenna; app abdominal appendages; lfw left forewing; lhw left hind wing; H head; MeTh mesothorax; MTh metathorax; mp mouthparts; PTh prothorax; rfl right foreleg; rfw right forewing; rhl right hind leg; rml right median leg; rhw right hind wing. Drawing by A.L. Scale bar, 10 mm.

- urn:lsid:zoobank.org:act:3802715B-4815-4DEB-A8B4-88782A44E48F
- *Type material.* Holotype MNHN.F.A71341 stored in the collection of the Muséum national d’Histoire naturelle, Paris.
- *Type stratum and age.* Pelitic Formation, “Red Permian”, Wordian, Middle Permian.
- *Type locality.* Gonfaron, Var, France.
- *Diagnosis.* As for the genus.
- *Description.* The trace fossil corresponds to a convex epirelief (possibly a counterpart), 43.1 mm long, 3.2 mm wide; with three distinct tagmata impressions; anterior tagma (H), 2.9 mm long and oval with a fine impressions ‘a’, curved forward, on top of ‘H’; thicker at its attachment points and tapering distally; at the bottom front of ‘H’, the impression ‘mp?’ elongated and curved upward in distal part; second tagma ‘Th’ 16.1 mm long, located behind ‘H’; ‘Th’ longer and thicker than ‘H’; ‘Th’ divided into three parts from front to back, their limits being determined by the positions of the legs and wings: first part, 3.2 mm long, appearing very short dorsally and carrying a pair of legs in its ventral part; second part, 7.3 mm long, very long and also carrying posteriorly a pair of legs and a pair of very long and wide structures (forewing tracks lfw and rfg); third part, 5.9 mm long, also very long, carrying one or two visible legs (only distal parts visible, their bases and femora being hidden by forewings) and a pair of hind wing tracks (lhw & rhw) quite distinct from forewing tracks, located just after level of bases of median legs; these wing structures being large, and covering at least 1/2 of total length of impression; third tagma impression ‘Abd’ the longest, 24.1 mm long, longer than ‘H’ and ‘Th’ combined, and of comparable thickness as ‘Th’, subdivided into a dorsal and a ventral parts; numerous indentations visible on it at fairly regular intervals (at least seven); no trace of dorsal or ventral appendages; a small structure ending in two points ‘app’ visible at apex of ‘Abd’.
- *Etymology.* Dedicated to the wizard Radagast of the mythology of J.R.R. Tolkien (*The Hobbit*, 1937) who tames stick-insects in the adaptation of P. Jackson (*The Hobbit: An Unexpected Journey*, 2012).

## Discussion

The impression most likely corresponds to a winged insect lying on its left side based on the presence of impressions of three tagmata with the anterior tagma much shorter than the two others and carrying structures that would correspond to antennae and possible mouthparts (mp) (Figs. 1a, 2); the median tagma corresponds to a thorax divided into a short prothorax bearing two legs, an elongated mesothorax with a pair of legs located anteriorly and a pair of wings located posteriorly, and a metathorax similar to the mesothorax, also carrying pairs of wings and legs; a segmented, elongated abdomen (Fig. 1c), legless and carrying a pair of complex terminal structures (cerci and/or ovipositor?) (Fig. 1b).

The ‘enlarged’ legs and inconspicuous joints between the thorax and legs indicate a certain viscosity of the substrate and possible weak motion of the legs. This could also be due to low movements of the substrate as indicated by the ripple marks. Most insects are fossilized in a dorsal or ventral position, especially paleopterans, but for the neopteran insects that hold their wings along the body, a lateral impression is possible. FBIs can remain unnoticed because of their shallow relief, requiring appropriate lighting to distinguish them. Here the delicate FBI is visible because the thin pelitic layer is exposed. The slab also has weak ripple-marks at the same level, showing a low-energy current and the presence of a biofilm^25–27^, which allowed the exceptional preservation of *Phasmichnus*. It is difficult to interpret the moment of life captured by this impression. This FBI probably corresponds to that of dying animal that was transported on fresh mud and laid its impression on it. The insect itself was probably destroyed later by the microbial activity. Evidence of such mats are frequent in the outcrop (folding of the mat, trace of grazing by organisms under the mat, etc.). Similar phenomena have been recorded in the late Jurassic lithographic limestone of Cerin, another well-known outcrop generated by microbial mats^28^.

No other known ichnotaxon of this size and shape can be brought closer to *Phasmichnus* gen. nov. Only Knecht et al.^5^ described an unnamed FBI of winged insect (attributed to an Ephemeropterida or a Plecoptera^29–31^) from the Late Carboniferous of the Massachusetts (USA), presenting an elongated body of comparable dimensions, but lacking distinct head and impressions of wings. It also preserves a possible cerci impression similar to that of *Phasmichnus*.

The position of the wings on the thorax and abdomen excludes the attribution of *Phasmichnus* gen. nov. to a Paleoptera because these have their wings unfolded over the body, except in the Diaphanopterodea, which have very different shorter and broader body shapes. The positions of the wing impressions of *Phasmichnus* exclude an attribution to the Ephemeropterida and Plecoptera and thus to the FBI described by Knecht et al.^5^. An Odonatoptera Archizygoptera or Zygoptera in which the wings may be lying on the body, especially in the case of a drowned individual with ‘wet’ wings, is conceivable. However, this attribution is unlikely because Odonatoptera have a highly modified thorax. Their short and reduced prothorax is followed posteriorly by a diamond-shaped structure as high as long and formed by the fusion of the meso- and metathorax^32^. *Phasmichnus* gen. nov. does not have a thorax of this type at all. We attribute it to a Neoptera capable of folding its wings over the abdomen. The presence of wings, the short prothorax plus the very elongated and narrow thorax and abdomen are only found in Phasmatodea among the extant insects, which also have the bases of their hind wings located behind the mesothoracic legs. This is indeed the case here. The other extant Neoptera have shorter abdomen and thorax in relation to their diameters, with the exception of the Mantophasmatodea, now apterous but whose ancestors were possibly winged. The extant and fossil Mantophasmatodea have the thoracic segments of nearly the same lengths, which is not the case here^33^. The Palaeozoic Caloneurodea also had narrow bodies but distinctly longer legs; and the Carboniferous Geraridae had an elongate prothorax and long legs, especially the hind legs that have enlarged femora, unlike *Phasmichnus* gen. nov^34^. The Late Carboniferous-Early Permian archaeorthopteran clade Cnemidolestidae had also elongate bodies with a relatively short prothorax, but they strongly differ from *Phasmichnus* gen. nov. in their very long and strong legs, especially the fore legs.

Extant stick insects, even the leaf-mimicking Phylliidae, differ from *Phasmichnus* gen. nov. in their shortened forewings, much shorter than the hind wings. But the Mesozoic winged representatives of the stem group Phasmatodea had fore- and hind wings of similar lengths, as in *Phasmichnus* gen. nov.^15,17^, possibly supporting its attribution to this lineage. Winged stick insects have their mid legs and forewings situated near the posterior margin of the elongate mesothorax as in *Phasmichnus* gen. nov.

Some molecular dating suggest that the Phasmatodea originated during the Middle Jurassic^35,36^, while recent paleontological discoveries show that the phasmid crown group was already well diversified at that time^15,17^. Triassic representatives of their stem group are known^14^, clearly more in accordance with more recent dating, viz. Permian-Triassic^37^, Middle Permian with confidence interval Carboniferous-end Permian^38^, or Carboniferous-Permian for stem group of Phasmatodea and Permian–Triassic for crown group^39^. The clade ((Mantophasmatodea + Grylloblattodea) + (Phasmatodea + Embioptera)) is considered as sister group of the Dictyoptera^40^, and therefore at least as old as the Carboniferous^41^. Thus, it is highly probable that representatives of the stem group of Phasmatodea or of the stem group of the clade ((Mantophasmatodea + Grylloblattodea) + (Phasmatodea + Embioptera)) existed in the Middle Permian. Furthermore, some Permian putative stem Embioptera have been described^42^, and an undescribed wing of an Embioptera was recently found in the Middle Permian of Southern China (Huang and Nel, in prep.), supporting the existence of stem group representatives of the two sister clades at that time. This would be in accordance to a putative attribution of the present discovery to the stem group Phasmatodea. *Phasmichnus radagasti* cannot be identified with certainty as a body impression of a stick insect sensu stricto as no anatomical features and no strict apomorphies of stick insects (e.g. fusions of first abdominal tergites and sternites with metathorax) are directly preserved, but it is an evidence of the presence of phasmatodean-like insects in the Middle Permian. A FBI is not a direct representation of the anatomy of the trackmaker: some anatomical structures may not have been imprinted and the overall impression may have undergone deformations, depending on the substrate^43^.

The typical morphology of Phasmatodea with narrow elongate bodies and elongate wings is clearly present in *Phasmichnus*. More precisely the general body and wing of *Phasmichnus* fits well with that of extant *Tropidoderus childerni* that has a very long body and hind wings and can hide itself in the vegetation with great efficiency (see internet site https://www.flickr.com/photos/petrichor/2177632362).

This type of morphology is consistent with adaptations to mimicry of elongated plant elements such as stems, branches or elongate leaves^43^, obviously present in the Permian vegetation and allowing concealing from predators. More generally, such narrow elongated body and wings are consistent with mimicry with plants among the extant terrestrial Neoptera (Mantodea, Heteroptera Reduviidae, Neuroptera Mantispidae, etc.), together with other functions such as predation.

The type of fossilization of *Phasmichnus* does not allow to find more information on this fossil. In particular, the possible pattern of coloration or details of ornamentations (spines, etc.), present in many extant stick insects and increasing the mimicry, are not available.

Several camouflages strategy are known among the late Carboniferous—Permian insects but they generally implicate disruptive strategies (spots and/or bands of different colors or even eyespots on wings). The archaeorthopteran family Cnemidolestidae shows an impressive diversity of such structures^45^. But plant mimicry is clearly less frequent during these periods. The orthopteran *Permotettigonia* is the only other case of a large leaf mimicry during the Middle Permian^24^. The strategy is different in *Phasmichnus* that would have imitated small branches and/or elongate leaves.

We found several slabs showing tetrapod tracks near *Phasmichnus radagasti*. The tetrapod ichnofauna of the type locality consists of small temnospondyl amphibians *(Batrachichnus salamandroides*), bolosaurian parareptiles (*Varanopus* isp.) and captorhinid eureptiles (*Hyloidichnus bifurcatus*). All these small vertebrates were probably insect hunters. *Permotettigonia* is the first accurate case of plant mimicry known in the Middle Permian of France^24^, *Phasmichnus* gen. nov. could be the second. The presence of a bio-mat could have been a large reserve of resources for the Hexapoda gathering on small water points and attracting the possible predators aforementioned. The trophic pressure of such potential predators in playa environments was possibly high enough to enable the rise of insects developing strategies of escape and/or plant-mimicry as early as the Middle Permian, in accordance with the opinion of Tihelka et al.^39^: ‘We recover a Permian to Triassic origin of crown Phasmatodea coinciding with the radiation of early insectivorous parareptiles, amphibians and synapsids’ [the only restriction to make to this sentence is that the Permian to Triassic stick insects did not belong to the crown but to the stem group of Phasmatodea]. Insectivorous synapsids and sauropsids, became very diverse in the Middle Permian^46^.

## Material and methods

### Preparation, observation and description

The holotype specimen was photographed with a Nikon D800 macro lens Micro Nikor 60/2,8 and drawn using Krita v.4.2.9 and PhotoFiltre 7 v.7.2.1. The nomenclature of Buatois et al.^10^ is followed to classify the types of arthropod tracks. The specimen was collected by one of us (RG).

### Geological setting

The specimen was collected in the Gonfaron A site, located south-west of the Luc Basin in the Pelitic Formation^47,48^. The Pelitic Formation corresponds to the upper part of the Luc Basin stratigraphy^47^. It is currently dated to the Wordian thanks to ichnological studies^47^. This formation is characterised by red pelites with drying up facies (such as mudcracks) and ripplemarks suggesting shallow non-marine environments^49^. The Gonfaron A site is located in pelitic badlands (locally named ‘red earth’) on the edge of the Maures plain and topped by a Triassic cliff (Bundsandstein). Sedimentological data from the site indicate a floodplain environment of playa type (see Supplementary Fig. 1 and Supplementary Information for a complete interpretation of the stratigraphy of the site). Several fossil arthropods have been found recently^50^. Others have been collected, also in the adjacent Permian basins and will be described later^51^.

## Supplementary Information

Supplementary Information 1.
