# Supplementary material for: A twig-like insect stuck in the Permian mud indicates early origin of an ecological strategy in Hexapoda evolution
Source: Sci Rep. 2021 Oct 21;11:20774. doi: 10.1038/s41598-021-00110-2 (PMC8531326; doi:10.1038/s41598-021-00110-2)
Supplement: Supplementary file 1 — Supplementary Information 1. [file 41598_2021_110_MOESM1_ESM.docx]

**A twig-like insect stuck in the Permian mud indicates early origin of an ecological strategy in Hexapoda evolution**

**Antoine Logghe^1,2,*^, André Nel^2,*^, Jean-Sébastien Steyer^1,**^, Valérie Ngô-Muller^2,3^ Jean-Marc Pouillon^2,4^ & Romain Garrouste^2,**^**

**Supplementary informations**

**Table 1. | Described full-body impressions of Carboniferous - Permian hexapods**

| **Period** | **Localisation** | **Age** | **Name (or reference)** | **Trackmaker** |
| --- | --- | --- | --- | --- |
| Permian | Antarctica (Transantarctic Mountains) | Permo-Triassic | *Orbiculichnus vulgaris* Holub & Kozur, 1981 | jumping insect or resting trace of an Euthycarcinoidea^1^ |
|  | Germany (Thuringia) | Early Permian | *Rotteroichnium* *longinum* Walter, 1983  *Avolatichnium* Walter, 1983  *Orbiculichnus vulgaris* Holub & Kozur, 1981 | landing trace of a Pterygota^2^  jump trace of an Archeognatha^2^  jumping insect or resting trace of an Euthycarcinoidea^2^ |
|  | New Mexico | Wolfcampian | *Tonganoxichnus robledoensis* Braddy & Briggs, 2002  *Hedriumichnus apacheensis* Braddy & Briggs, 2002  *Rotteroichnium major* Braddy & Briggs, 2002 | Monura (Hexapoda Archaeognotha)^3^  Ephemeroptera (Palaeoptera, Pterygota) or Plecoptera  (Neoptera, Pterygota)  adult Odonatoptera or Palaeodictyoptera |
| Carboniferous | Kansas | Gzhelian | *Tonganoxichnus buildexensis* Mángano *et al*., 1997 | Monura (Hexapoda Archaeognotha)^4^ |
|  | Massachussetts | Late Carboniferous | Knecht *et al*., 2011 | Ephemeropterida^5^ |

**Detailed stratigraphy of the type locality at Gonfaron (Supplementary Fig. 1)**

The Gonfaron A locality is predominantly composed of red pelites. However, to better understand the different steps of deposition and thus, the evolution of the palaeoenvironments, fourteen sequences have been identified. Notice that the numbering (1 to 14) is done in relation to the outcrop and not in relation to the overall formation (which is still under description). The difficulty of marking the sequences in the pelite layers prevents a precise localisation inside the Pelitic Formation, essentially due to tectonic accidents and possible lateral variations.

1 – The base of the sequence I is unknown because of the presence of the road through the outcrop. The presence of rhizomes indicates a zone of exondation.

2 – The sequence II is characterized by channels, indicating a deltaic or fluvial input. These channels, eight in number, are intercalated within decimetric layers of red pelites. The light variation of granulometry in these channels suggests a deposit under a low height of active water. The last channel is of about three metres and thirty centimetres large, with an abundance of fossil traces (*Scoyenia*). The sequence ends with a decimetric thickness of red pelites characteristics of a floodplain. It is topped by a layer of indurated pelites with mudcracks, body impressions of *Triops* and arthropod trackways: these ichnofossils characterise a possible exondation.

3 – A metric layer of red pelites begins the sequence III. The metric thickness of the layer of red pelites suggests a regular and constant fine sediment input. A decimetric layer of red pelites with rhizomes is characteristic of an exondation at the top of the sequence.

4 – The sequence IV begins with sixty centimetres of red pelites and ends with an alternation of red pelites and more indurated thin greyish levels. The presence of five levels of mudcracks clearly characterises emerging facies.

5 – We preferred to separate sequence V from sequence IV, even if they could have been associated. Sequence 5 presents a layer of 80 cm of pelites and ends with a more indurated and thin greyish levels of pelites with mudcracks.

6 – The sequence VI begins with a layer of red pelites of about 3 m and ends with a layer of indurated argilites in laminated plates characterised by the presence of mudcracks as well as ripple marks and raindrops. There are also abundant ichnites of bio-mats as well as arthropod trace fossils (*Scoyenia*, *Planolites* and *Acripes multiformis*) and trackways (*Lithographus hieroglyphus*). Several tetrapod trackways such as *Hyloidichnus major*, *Hyloidichnus* isp, *Microsauripus/Varanopus* sp.*, Dromopus* sp. and *Batrachichnus salamandroïdes* have been recovered from sequence VI. Finally, cone ichnofossils are present. This assemblage of ichnofossils characterises a facies close to exondation and a possible ephemeral lake of decametric extension, attracting an abundant fauna. This is where *Phasmichnus radagasti* gen. et sp. nov. has been recovered.

7 – The sequence VII, less thick than sequence VI, is composed of a metric layer of red pelites and finishes by a level of indurated thin greyish levels of pelites with layers of mudcracks. Tetrapod footprints have been recovered in this sequence. This level is topped by a layer composed of an alternations of red pelites and thin ‘yellowish’ levels.

8 – Sequence VIII is mainly composed of red pelites and ends with a level of indurated pelites bearing of mudcracks.

9 – Sequence IX is a small pelitic sequence finishing with thin ‘yellowish’ levels with mudcracks; body impressions of *Triops*, arthropod trackways, and plant remains.

10 – Sequence X is of about three metres thick, composed at its base of alternations of red pelites and thin ‘yellowish’ layers, 2-5 cm thick. No fossil traces have been found in these layers. The sequence ends with a level of mudcracks with arthropod traces and ichnites of bio-mats.

11 – Sequence XI begins with a metric layer of red pelites with an abundant presence of bioturbations (*Scoyenia*). The sequence ends with a thin ‘yellowish’ layer in plates, 15 cm thick.

12 – Sequence XII is a pelitic assemblage, 2.5 m thick. The transition with sequence 13 is marked by a change in the induration of the pelites.

13 – Sequence XIII is also a pelitic assemblage, ca. 2 m thick, with bioturbations in upper part (*Scoyenia*).

14 – Sequence XIV is incomplete, mainly composed of red pelites.


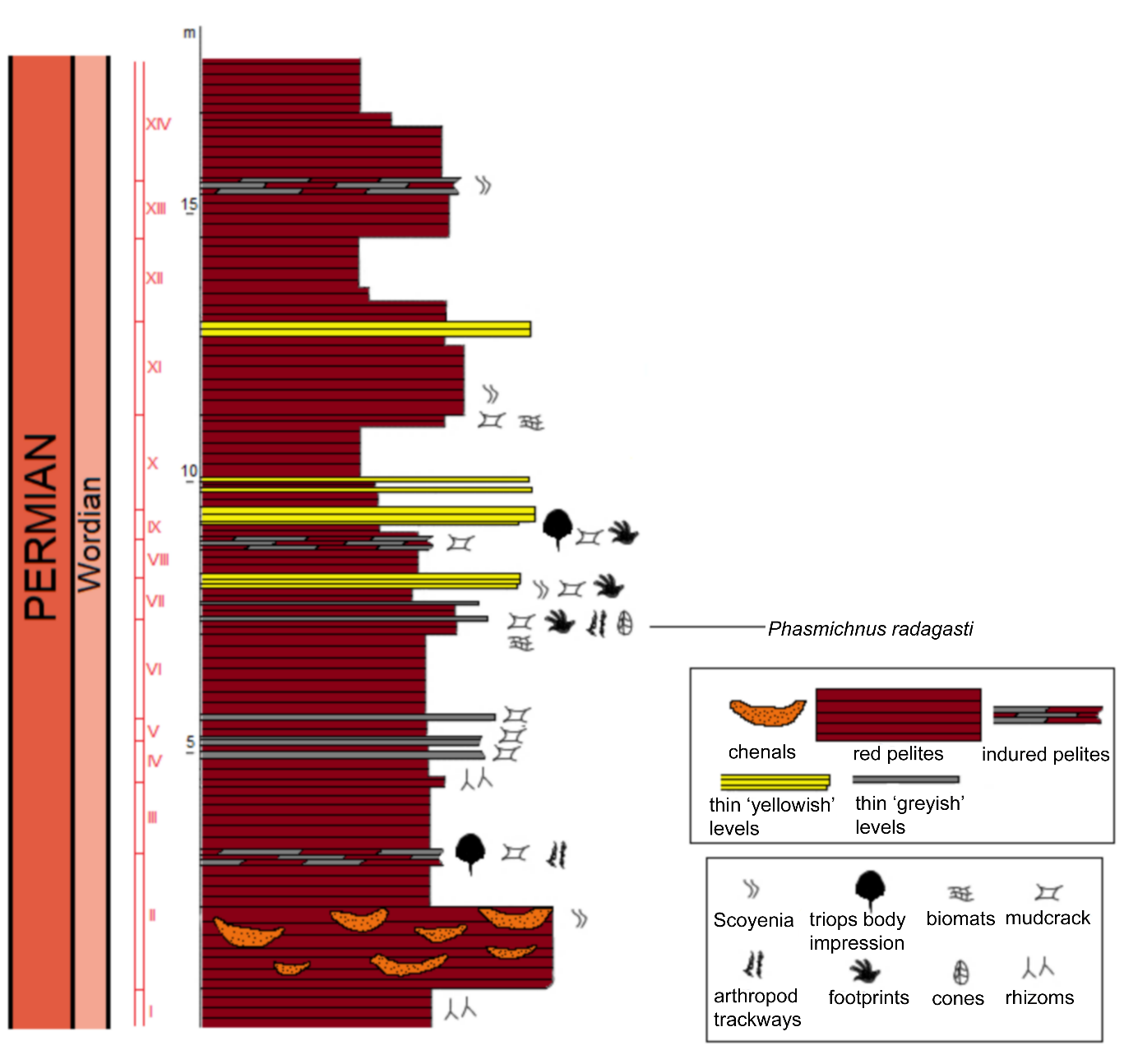


**Supplementary Fig. 1 | Stratigraphy of the type locality**. Middle Permian of France (Var, Gonfaron). Red scale indicates the sequences of deposition identified (see description in Supplementary information). The proportionality and thickness of the sequences are preserved, however, for clarity concerns, some levels inside of the sequences have been exaggerated.

**References**

1. Holub, V. & Kozur, H. Arthropodenfährten aus dem Rotliegenden der CSSR. *Geologisch-Paläontologische Mitteilungen Innsbruck* **11**, 95-148 (1981).
2. Walter, H. Zur Taxonomie, ökologie und biostratigraphie der ichnia limnisch-terrestrischer arthropoden des mitteleuropäischen jungpaläozoikums. *Freiberger Forschungschefte C* **382**, 146-193 (1983).
3. Braddy, S.J. & Briggs, D.E.G. New lower Permian non-marine arthropod trace fossils from New Mexico and South Africa. *Journal of Paleontology* **76**, 546-557 (2002).
4. Mángano, M.G., Buatois, L.A., Maples, C.G. & Lanier, W.P. *Tonganoxichnus*, a new insect trace from the Upper Carboniferous of eastern Kansas, *Lethaia* **30**, 113-125.
5. Knecht, R.J., Engel, M.S. & Benner, J.S. Late Carboniferous paleoichnology reveals the oldest full-body impression of a flying insect. *Proceedings of the National Academy of Science* **108**, 6515-6519 (2011).
